# Wanted, an Anthrax vaccine: Dead or Alive?

**DOI:** 10.1186/1476-9433-4-5

**Published:** 2005-04-18

**Authors:** Kendall A Smith

**Affiliations:** 1 The Division of Immunology, Department of Medicine, Weill Medical College, Cornell University, New York, NY, 10021, USA

## Abstract

It has been more than 100 years since the realization that microbes are capable of causing disease. In that time, we have learned a great deal as to how each organism has adapted to the immune system so as to avoid elimination. As well, we have also learned an immense amount since Louis Pasteur first proposed that the solution to infectious diseases was to culture the microbes and attenuate their virulence, so as to use them as vaccines. From the optimism and promise of the 19^th ^century and immunization as the ultimate answer to the invasion by the microbial world, to the scientific realities of the 21^st ^century, it is of interest to retrace the steps of the earliest microbiologists cum immunologists, to realize how far we've come, as well as how far we yet have to go. This editorial focuses on the history of anthrax as a microbial disease, and the earliest efforts at producing a vaccine for its prevention.

## Editorial

"In France, one can be an anarchist, a communist or a nihilist, but not an anti-Pastorian. A simple question of science has been made into a question of patriotism."

*August Lutand*

*"Pasteur et la Rage"*

*1887*

" [Pasteur] was the most perfect man who has ever entered the kingdom of science."

*Stephen Paget*

*"The Spectator"*

*1910*

To continue the series of ***"The Classics of Immunology" ***we turn next to Louis Pasteur's development of the live attenuated anthrax vaccine, first published in 1881 [1]. In this publication, Pasteur claimed that he could attenuate the anthrax organism by simply culturing it in air at elevated temperatures. Together with his announcement a year previously of a vaccine for chicken cholera [2], he thus introduced the concept of attenuating the virulence of organisms so that they could be used as live vaccines. Audaciously, he speculated that all microbial diseases could be prevented this way: one only had to learn how to culture the offending agent. Of course we now know that it is not quite as simple as Pasteur originally claimed, particularly for bacterial infections. A brief review of this interesting period in early immunology is warranted before one can fully appreciate our present day knowledge.

When the anthrax spores were sent through the mail soon after the terrorist attacks on 9/11/2001, I was perplexed. I really had no idea what disease, or diseases, that were caused by the anthrax microbe. I vaguely recalled from my medical school days that the anthrax microbe is a Gram-positive rod. I also recalled that Louis Pasteur had developed a vaccine against anthrax, but that was about all that I could remember. Accordingly, I consulted my microbiology texts and went on-line, to see what I could learn.

From my texts I learned that the anthrax organism has the unusual property of undergoing a metamorphosis from a multiplying vegetative bacteria into a *spore *(from the Greek, *sporos *= seed), which is defined as an inactive resting or resistant form produced within the body of a bacterium.

I also learned that anthrax was the first disease actually proven to be caused by a microbe, by Dr. Robert Koch, who reported on his experimental findings in 1876 [3]. This piece of information really piqued my interest, because I had no idea that anthrax was so central to the history of medicine. Like most other physicians of my generation, I had never seen nor heard of an actual case of anthrax. However, I did know that anthrax was often mentioned as a possible bio-weapon that could be used by terrorists, but I did not know why it was so dangerous, or exactly what symptoms were produced by the infection.

I remembered visiting one of my friends at the Frederick Cancer Research Facility (FCRF) in Frederick Maryland in the 1970's. The FCRF was originally Fort Dietrich, which from 1943 to 1969 was the U.S. Army base devoted to chemical and biological warfare research. My friend pointed out a large 5–6-story building that had all of the windows and doorways covered over with concrete blocks. This building had housed all the anthrax research, and it was still contaminated by anthrax spores, which were extremely deadly, so that the building had been hermetically sealed, rather than razed, when President Nixon discontinued chemical and biological warfare research. As far as I know, that building is still there in Frederick.

To learn more about Robert Koch and anthrax, I turned to a book by Eli Metchnikoff, published originally in 1905, entitled **"The Founders of Modern Medicine: Pasteur, Koch, and Lister" **[4]. Metchnikoff was a Russian zoologist, who observed the phenomenon of phagocytosis for the first time, while studying starfish larvae off the coast of Naples in the 1870's [5]. Subsequently, in 1885, he was recruited by Pasteur to become the first *Chef de Service *at the newly formed Institut Pasteur in Paris, where he championed the idea that cells actually are responsible for immunity.

Beginning with Ignaz Semmelweis' observations on the possible cause of puerperal fever (childbed fever) in 1850 [6], the notion that small microscopic living things might cause disease and death began. Then, Louis Pasteur's demonstration in 1857 that fermentation of lactic acid into alcohol and carbon dioxide was actually caused by living organisms (animal infusoria), and not by the then popular theory of "spontaneous generation", set the stage for the importance of microbes in everyday processes [7]. Subsequently, Joseph Lister's 1867 descriptions of the use of antiseptics in the practice of treatment of compound fractures and at surgery [8,9], promoted a growing notion that removal of microbes isolated from wounds and other degenerative tissues could improve the outcome of the patient. However, the most common belief still held was that any microbes found in suppurating tissues were the result and not the cause of the fetid, morbid state. The morbidity was thought to arise spontaneously via chemical reactions. Any association with living microbes was considered fortuitist.

In the words of Metchnikoff,

"A powerful impulse was necessary to change this inchoate idea of organized (chemical) ferments into a rigorously proven scientific truth that microbes were responsible (for putrifaction and disease). Robert Koch started such an impetus in his 1876 paper on anthrax. This young health officer in the little city of Wolstein, a god-forsaken hole in Posen (Prussia), suddenly came into the limelight of science. His work was indeed a model of true scientific creativeness. Living in a region in which anthrax was endemic, he set about to study it, without the help of laboratory or library, and was always thrown back on his own resources. He worked in his own rooms where for lack of gas illumination he was obliged to use a petroleum lamp. By means of plates covered with moist sand he constructed a semblance of an apparatus for growing cultures of bacteria. Nevertheless, he achieved results superior to anything yet accomplished. He was the first to succeed in changing the thread-like microscopical corpuscles identified by others (in France) into identifiable filaments (chains of rods) and then into beads consisting of minute grains, the spores. This great discovery of the spore of anthrax removed all doubts regarding the role of bacteria in the causation of anthrax, for it illuminated all points hitherto left unexplained."

Throughout medieval times, anthrax was a disease primarily of livestock, and it still is considered so, which explains why I was unfamiliar with it. In humans, the most common affliction is a skin inflammation that matures into a very characteristic ugly black eschar, from whence the disease was named from the Greek: anthrax = *coal, carbuncle*. In the 19^th ^and early 20^th ^centuries, cutaneous anthrax was also known as "wool sorters disease", because farmers and woolen workers would contract it from handling animals and wool contaminated with anthrax spores. For the livestock industry, anthrax was a serious problem, in that many animals would succumb to a more severe disease manifested by both gastrointestinal and pulmonary symptoms. Once animals died and their corpses were allowed to disintegrate in a pasture, it was well known that the particular pasture was thereafter suspect, in that the reintroduction of fresh animals in the spring often resulted in the reappearance of the disease. Thus, as a result of Koch's experiments, it was realized that the ability of the microbe to sporulate enabled it to withstand the harsh temperatures and conditions that often occur during the winter months.

Nowadays the livestock industry is protected from anthrax by vaccination. This protection of farm animals extends to farmers and other humans, such as textile workers and vets, so that anthrax infection of humans has become exceedingly rare, especially since the time of Koch. However, what is the situation with an anthrax vaccine for humans? In our country, the only vaccine available is only being administered to soldiers. Since the postal anthrax scare of 2001, the Administration, via the Defense Department ordered all new recruits to receive the vaccine. However, the vaccine is reportedly not 100% effective, requires 6 injections over a period of a year and a half, and is associated with side effects/toxicities that have led some army personnel to refuse it.

Louis Pasteur introduced a live attenuated anthrax vaccine more than 100 years ago. So why is the currently used vaccine so cumbersome and toxic? Also, is the current vaccine similar to the one introduced by Pasteur? Is the vaccine that is used for animals the same as the one used for humans?

When contemplating these questions, I remembered that in 1998, while in France, I happened to read an article in Le Figaro, which announced that the anthrax vaccine introduced by Pasteur in 1881 was in fact not the live attenuated vaccine that Pasteur had suggested he used at the time. Instead, the vaccine was a chemically killed vaccine that had been developed and introduced by one of Pasteur's rivals, a Dr. Toussaint, who was a veterinarian from Toulouse, France.

To understand the implications of the announcement by a leading French newspaper that the icon of the French scientific accomplishment and integrity had committed what amounts to scientific fraud, it is necessary to research the source documents of Pasteur's experiments and publications.

After Pasteur, the chemist, had dispensed with the theories of "spontaneous generation" as responsible for the chemical changes responsible for fermentation of sugar into alcohol in 1857, he went on over the next 20-years to perform a series of careful microbiological experiments in applied science in studies of bacterial contamination confronting the silk worm industry, as well as the wine, vinegar and beer industries, thereby establishing the importance of microbes for everyday endeavors. In the process of doing so, he became almost deified in France, if not the rest of the world as the icon of a scientist.

Thus, in April of 1878, just two years after Koch's revolutionary publication proving the microbiological cause of anthrax, Pasteur presented a "Summary" to the Academy of Sciences, essentially claiming priority of the germ theory of disease [10]. According to Pasteur:

"The only way currently available to science to experimentally prove that a microscopic organism is the cause of both the illness itself and its transmission, is to subject the microbe to serial cultures."

Pasteur then goes on to describe his experiments with the anthrax bacillus, never mentioning that Koch had already demonstrated the culture of the anthrax microbe two years earlier. In concluding, he states that:

*"I ask the Academy not to dismiss these curious results before I demonstrate one important theoretical conclusion. We insist on demonstrating at the start of these studies *(***that are opening a whole new world of knowledge***) *a proof that the cause of transmissible, contagious and infectious diseases resides essentially and uniquely in the presence of microorganisms."*

Not yet two years later, in February of 1880, Pasteur again presented to the members of the Academy a treatise entitled "*Of Infectious Diseases, Especially the Diseases of Chicken Cholera" *[2].

In this presentation, Pasteur first reminded the members that the theory of spontaneous generation was false, as demonstrated by his very own experiments performed more than 20 years previously. He then set the stage by stating:

"Infectious diseases consist of most of the major disasters, such as smallpox, scarlet fever, rubella, syphilis, glanders, anthrax, yellow fever, typhus, and bovine plague."

Pasteur then discussed the phenomenon of vaccination as introduced by Sir Edward Jenner almost 100 years before as something already known by the common man, and essentially claims immunity for all other microbes for himself:

"*The practices of vaccination and variolization have been known in India for the longest time. Even before Jenner demonstrated the efficacy of vaccinia, people of the countryside where he practiced already knew that cowpox protected against smallpox. The facts about vaccinia are unique, but the facts about nonrecurrance of virulent diseases are more general. The organism never expresses twice the effect of chicken pox, scarlet fever, typhus, plague, smallpox, syphilis and others, as the immunity lasts for a long time at least."*

Pasteur then introduced the problem of chicken cholera, and mentioned that M. Toussaint, a professor at the veterinary school of Toulouse had been the first to culture and isolate the microbe that he thought to be responsible for the cause of the disease of chickens. Pasteur went on to say that he had discovered an improved culture medium for the microbe, and....

"We can diminish the microbe's virulence by changing the mode of culturing. This is the crucial point of my subject. I ask the Academy not to criticize for the time being, the confidence of my proceedings that permit me to determine the microbe's attenuation, in order to save the independence of my studies and to better assure their progress."

With this presentation to the Academy, Pasteur merged the science of microbiology with that of what subsequently became known as immunology for the first time. As well, this presentation to the public revealed a crucial aspect of Pasteur's experiments and thinking as to his perception of the importance of his findings. In France at the time it was common practice to submit a sealed note (called a *pli cachete*) on an important scientific discovery to the Academy of Sciences to secure or protect one's priority. By comparison, an official patent application (*brevet d'invention*) was necessary to establish one's right to the commercial exploitation of that discovery. Pasteur thus kept it a secret as to exactly how he had attenuated the virulence of the chicken cholera microbe for more than 9 months, until October of 1880.

Eventually, Pasteur disclosed that his methods simply involved culturing the microbe exposed to atmospheric oxygen for prolonged intervals, i.e. longer than 2–3 months. However, he never explained why oxygen should weaken the microbe's virulence, especially as the chicken cholera microbe is an aerobic organism. It is likely that he did not want to risk others trying to repeat his methods, both from the standpoint of the fear of their success as well as their failure.

Pasteur then described using the "live attenuated" cholera vaccine to immunize animals against lethal challenges of the microbe, and stated that

*" It seems as if the initial microbe inoculations *(of the live attenuated vaccine) *have ****depleted ****a certain element that healing does not reconstitute and that the absence of which hinders the development of this small organism (*when re-inoculated a second, third, and fourth time). ***This explanation will without doubt, become general and applied to all infectious diseases.***

*I would like to point out to the Academy two main consequences to the facts presented: ****the hope to culture all microbes and to find a vaccine for all infectious diseases that have repeatedly afflicted humanity, and are a major burden on agriculture and breeding of domestic animals." ***

The importance of Pasteur's theory, i.e. that it was possible to attenuate the virulence of *all *microbes, simply by passing them in special culture conditions can only be appreciated by understanding the competition that developed between Pasteur and Toussaint in the summer of 1880 involving different approaches to the creation of a vaccine for anthrax.

Pasteur had begun working on a vaccine for anthrax 3 years previously, soon after Koch's announcement on the isolation of the causative anthrax bacillus [11]. On July 12, 1880, Henri Bouley (a fellow veterinarian and friend of Toussaint) read before the Academy of Sciences a report from Toussaint (who was not a member of the Academy), which described the initial results of his experimental vaccine trials. In contrast to Pasteur's "live attenuated" vaccine, Toussaint generated his vaccine simply by killing the bacilli by heating for 10 minutes at 55°C. Using this vaccine, Toussaint had conducted trials on 8 dogs and 11 sheep. Of the 8 dogs, 4 had been injected with the vaccine and had survived a series of 4 successive injections of virulent live anthrax. By comparison, all 4 unvaccinated dogs succumbed to the first injection. A similar result was obtained with the sheep.

In August, while vacationing, Pasteur heard the news of Toussaint's vaccine experiments from Bouley. He responded as follows [11]:

*"My very good colleague*,

Since yesterday morning, when I received your letter, the extracts of the journals, and the Summary of the Academy of Sciences-all at the same time -I have been in astonishment and admiration over the discovery of M. Toussaint-in admiration that it exists, in astonishment that it can be. It overturns all the ideas I had on viruses, vaccines etc. I no longer understand anything. Ten times yesterday, I had the idea of taking the train to Paris. I really cannot believe this surprising fact until I've seen it, seen it with my own eyes, though the observation that establishes the fact makes me want to confirm it to my own satisfaction.

The Academy of medicine has thus received a severe lesson. It will surely have grasped that one does not deal lightly with facts of this order in public, that contemplation is appropriate in the face of such solutions to such problems.

I am too moved to write more fully. I have dreamed about it, both asleep and awake, all through the night.

Best to you and thanks.

L. Pasteur

Pasteur's expression of surprise and agitation makes sense only in the context of his general theoretical views on diseases and immunity. Because of his successes in his studies of the metabolism of living microbes, Pasteur naturally extended his concepts to immunity. Linking immunity with the biology of microbes, especially the nutritional requirements of the virulent microbe, he suggested that the tissues of the invaded host might contain only trace amounts of some nutrients required for the growth and survival of the microbe, just as some culture media contained only trace amounts of vital nutrients. If so, the invading microbe might soon exhaust the supply of these trace substances, rendering the host an unsuitable medium for the microbe's subsequent cultivation. Thus, the host would not support the growth of a subsequent infection by the virulent microbe, and would be "immune" (Latin, *immunis*; free, exempt). Also, an attenuated microbe would be one that had been stressed by cultivation either *in vitro *or *in vivo *in an environment that was limiting in essential nutrients, thereby somehow loosing its virulence.

Thus, central to Pasteur's conception of immunity, was the biological activity of a living, if attenuated, microbe that **depleted **the host of essential nutrients. It was Toussaint's claim that he had in fact produced a "dead" vaccine against anthrax that moved Pasteur to state that "it overturns all the ideas I had on viruses, vaccines, etc."

As one might imagine, given Pasteur's theory, and his statements already made to the Academy, his lance had been planted. He could not, and would not, graciously admit that he was wrong. The story only goes downhill from this point. In the public critique that Pasteur was soon to issue against Toussaint's work, his central theoretical concern was precisely the question of "live vs. dead" vaccines.

In August, 1880, soon after announcing his heat killing method of vaccine production, Toussaint switched his procedures and had begun to subject the bacilli to the action of carbolic acid, which had long been used as a disinfectant and had more recently become famous as Joseph Lister's "antiseptic" of choice for the treatment of surgical wounds.

Pasteur did not announce the discovery of his own "live attenuated" anthrax vaccine until February 28, 1881. Of significance, Pasteur linked his new vaccine with his earlier chicken cholera vaccine by ascribing attenuation in both cases to the action of atmospheric oxygen. However, there was an important difference between the production methods of the two vaccines. Unlike the chicken cholera microbe, the anthrax bacillus formed spores that *"resisted the attenuating effects of atmospheric oxygen"*. It had taken much time and effort to ascertain that a spore-free culture of anthrax could be produced at a temperature of 42°–43°C.

Subsequently, on March 21^st^, Pasteur reported further successful results testing his vaccine in sheep, which stimulated a challenge by a veterinarian, Hippolyte Rossignol from Pouilly-Le-Fort, to test the new vaccine at his farm in Melun, 40 kilometers from Paris. Examination of Pasteur's lab notebooks [11] reveals that he had been conducting small trials, testing his vaccines in animals during this time, with less than conclusive results as to the protective efficacy of the live atmospheric oxygen attenuated vaccine. However, at the same time, Pasteur's lab was testing a vaccine prepared by M. Chamberland, who was experimenting with a "dead" vaccine prepared by chemical treatment with potassium-bichromate. In small-scale tests this vaccine was working.

If Pasteur had failed to accept Rossignol's challenge, he would certainly have damaged his priority competition with Toussaint. Moreover, there were already rumors that Pasteur was really seeking to profit financially from his "secret remedies" against livestock diseases. Therefore, Pasteur "impulsively" accepted the challenge and on April 28, 1881, and he signed a detailed and demanding protocol, which was performed in May.

There is a wonderfully detailed accounting of the drama of the public trial that Pasteur publicly presided over on June 2, 1881 [12]. There were more than 200 observers, including government officials, local politicians, veterinarians, farmers, agriculturists, cavalry officers and newspaper reporters. Of 50 sheep in the trial, half were vaccinated on May 5^th ^and May 17^th^, while the other half served as unvaccinated controls. All of the sheep were then challenged with a virulent culture of anthrax bacilli on May 31st. Just 3 days later, all of the vaccinated sheep were alive, while most of the unvaccinated sheep were already dead, with the remaining obviously very ill.

Only Pasteur and his collaborators knew of the real nature of the vaccine used for this famous trial. Pasteur had not used the live attenuated vaccine that he had emphasized was so important for his success with chicken cholera. Instead, the "dead" vaccine of Toussaint prepared by Chamberland by treatment with potassium-bichromate was used [11].

The up-shot of this public demonstration of Pasteur's vaccine was that he received credit for developing the first successful vaccine against anthrax. Toussaint subsequently published only 2 more scientific papers before he died in 1890 at the age of 43, after suffering a mental breakdown [11]. It was not until 1998, that the French government officially recognized Toussaint's vaccine as the first effective vaccine against anthrax.

It is noteworthy that Robert Koch, who became one of Pasteur's chief competitors, hailed Toussaint as the worthy inventor of vaccination against anthrax, and persistently denigrated Pasteur's contributions to microbiology [4].

There are many other questions that remain unanswered, such as the nature of the vaccine that Pasteur's laboratory supplied to the many people who requested doses for their animals. Parenthetically, it is noteworthy that the vaccine was manufactured commercially by Pasteur's team, and yielded a substantial income for the new Pasteur Institute, which was initiated four years later, in 1885 [11]. As well, what of the others in the Pasteur group, all of who knew of the real nature of the vaccine that was used at Pouilly-Le-Fort? Probably of most importance, what became of the concept of attenuating microbes by exposing them to atmospheric oxygen? Surely, all competent bacteriologists who worked in the early and mid 20^th ^century had to know that Pasteur had been wrong, and that it was impossible to attenuate aerobic microbes by simply culturing them in the open air. Why was this not aired?

Fast-forwarding to the present, I have asked Julia Wang and Michael Roehrl to bring us up-to-date on the present state of the art of anthrax vaccine research. As detailed in their excellent review, the nature of the anthrax vaccine that is in use presently to immunize at-risk wool mill workers, veterinarians, laboratory workers, livestock handlers, and members of the Armed Service is a cell-free filtrate. The vaccine was developed in the 1950s and 1960s for use in humans and was licensed by the FDA in 1970. It has undergone extensive testing in monkeys and has been found to be effective in protecting against pulmonary anthrax after an experimental aerosol challenge.

The remarkable virulence of anthrax, which makes it such an attractive microbe for bio-warfare, resides in several unique features, including its capability to sporulate, thereby surviving extremes of the environment, its capsule, which impairs phagocytosis, and its toxins, as well as how the toxins interact with and eventually incapacitate the immune system. A lot has changed since the days of Pasteur and Toussaint. We are fortunate that now we have a better understanding of bacteria in general and the anthrax bacillus in particular. Now it is possible to make a safe and effective vaccine for such a virulent organism, based not on a live attenuated vaccine as proposed by Pasteur, but a vaccine more like the inactivated preparation originally developed by Toussaint.

Access to the papers referred to in this editorial can be obtained at [13]
